# EpitoCore: Mining Conserved Epitope Vaccine Candidates in the Core Proteome of Multiple Bacteria Strains

**DOI:** 10.3389/fimmu.2020.00816

**Published:** 2020-05-05

**Authors:** Tayna S. Fiuza, João P. M. S. Lima, Gustavo A. de Souza

**Affiliations:** ^1^Bioinformatics Multidisciplinary Environment, Universidade Federal do Rio Grande Do Norte-UFRN, Natal, Brazil; ^2^Department of Biochemistry, Universidade Federal do Rio Grande do Norte-UFRN, Natal, Brazil

**Keywords:** reverse vaccinology, pangenome, prokaryotes, epitope prediction, vaccine candidates, bioinformatics

## Abstract

In reverse vaccinology approaches, complete proteomes of bacteria are submitted to multiple computational prediction steps in order to filter proteins that are possible vaccine candidates. Most available tools perform such analysis only in a single strain, or a very limited number of strains. But the vast amount of genomic data had shown that most bacteria contain pangenomes, i.e., their genomic information contains core, conserved genes, and random accessory genes specific to each strain. Therefore, in reverse vaccinology methods it is of the utmost importance to define core proteins and core epitopes. EpitoCore is a decision-tree pipeline developed to fulfill that need. It provides surfaceome prediction of proteins from related strains, defines core proteins within those, calculate their immunogenicity, predicts epitopes for a given set of MHC alleles defined by the user, and then reports if epitopes are located extracellularly and if they are conserved among the core homologs. Pipeline performance is illustrated by mining peptide vaccine candidates in *Mycobacterium avium hominissuis* strains. From a total proteome of ~4,800 proteins per strain, EpitoCore predicted 103 highly immunogenic core homologs located at cell surface, many of those related to virulence and drug resistance. Conserved epitopes identified among these homologs allows the users to define sets of peptides with potential to immunize the largest coverage of tested HLA alleles using peptide-based vaccines. Therefore, EpitoCore is able to provide automated identification of conserved epitopes in bacterial pangenomic datasets.

## Introduction

The characterization of specific molecular targets for controlling and removing bacterial infections is an important and challenging task. Surface proteins are key molecules for infection initiation and are at the interface with the host immune system (1). Furthermore, they are underrepresented in many experimental studies due to the fact that transmembrane proteins are heterogeneous, hydrophobic, and often detected at low abundance (2). Conventional screening of antigens in surface proteins is laborious, expensive and time-costly.

*In silico* approaches became a desirable method for mining candidate antigenic proteins. It has been largely employed to characterize single or sets of sequences of interest (3–5). Reverse vaccinology (RV) approaches use bacterial genomic information to achieve large scale antigen classification, and often integrate diverse levels of molecular prediction, such as subcellular localization, membrane adhesion and human cross-reactivity [for a review, see (6)]. RV was first established to investigate antigens in serogroup B meningococcus (7) and has been employed for many different pathogens [for reviews, see (8–10)].

Not surprisingly, many bioinformatics tools were developed in the past decade to facilitate epitope prediction in large datasets. They are either: (i) decision-tree approaches, i.e., a large list of protein sequences from the organism of interest is submitted to the tool, which then apply different filters under specific parameters to trim the list to a final, smaller dataset of potential vaccine candidates; or (ii) machine-learning approaches, which classify epitope candidates based on rules created by a training set of known, well-characterized epitopes. Dalsass et al. had recently performed comparisons and evaluations of six of the most used, publicly available reverse vaccinology tools (6). Such pipelines, however, are limited to the analysis of a single proteome of a bacterial strain. Vaxign [http://www.violinet.org/vaxign/ (11)] allows multiple comparisons of strains in its web-server interface, however only a fraction of strains with complete sequenced genome are available for analysis.

As more genome information of multiple strains of the same species was made available, the presence of a core and accessory genome was characterized in several of those species (12). Only recently such genomic features are being taken into consideration when performing RV, as has been shown for *Helicobacter pylori* (13), *Acinetobacter baumanii* (14), *Leptospira interrogans* (15), pathogenic *Brucella* spp. (16), and *Corynebacterium pseudotuberculosis* (17). While these analyses provided the complete decision-tree performed, none of them provided the in-house scripts used for integration of all tools employed in their approaches. To our knowledge, the only tool available for pangenomic analysis and RV prediction is PanRV (18), which employs routinely used membrane and subcellular localization predictions. It also combines additional filters to enrich possible vaccine candidates such as gene essentiality and/or virulence factor predictions.

PanRV (and other RV tools) are protein-centric, i.e., intact proteins are reported as vaccine candidates. If a vaccine is developed based on intact proteins, it could be argued that proteins containing more than two transmembrane domains are poor vaccine candidates due to the difficulty to purify them. However, synthetic peptides-based vaccines had been largely used in vaccine development in recent years. They offer many advantages to integral proteins purified from the pathogen, such as: (i) fully *in vitro* manufacture, with less chance of biological contamination from pathogen; (ii) full characterization as a chemical entity; (iii) higher stability and storability; (iv) smaller chance to induce non-specific reactions in the host [for a review, see (19)].

In a peptide-centric analysis, even difficult to isolate proteins with highly immunogenic peptides could be considered for vaccine design. Based on this reasoning, we developed EpitoCore, a bioinformatic strategy that integrates surfaceome and subcellular localization prediction to pangenomic characterization, and further defines conserved epitopes in core proteins. For transmembrane proteins, EpitoCore correlates structure topology and epitope position to guarantee prediction of valid epitopes exposed to extracellular side.

## Methods

### Scripts Design and Format

In-house scripts were created using Python version 3. CMG Biotools scripts are written in Perl and we added two modifications to guarantee that: only the best alignment to a query sequence is reported instead of a list containing all alignments within the requested parameters; and that homologs are selected only if a bidirectional best hit criteria is fulfilled (see below). The shuffle script from the package BBMAP (https://www.osti.gov/biblio/1241166) ordered the multifasta files so fasta_ids and CMG Biotools hash ids could be paired. The TMHMM script was created using Perl as well. PSORT-B is executed independently through a command line interface. IEDB peptide-HLA binding affinity predictors are written in Python 2.7. Final scripts for immunogenic analysis and image production were created using R studio version 1.1.442.

Users must provide a text file containing the species or the “intraspecies” name (as seen in the NCBI's assembly summary information) to be investigated as input to the script get_proteome.py. This script outputs a comma-separated file with the assembly summary information requested and downloads the protein sequence datasets (.faa files) to a user' specified folder. The .faa files are used independently for transmembrane and cell localization prediction. Script predict_transmembrane.py will call the TMHMM script, output the whole prediction in a folder, and filter proteins classified as transmembrane. Users must install and execute PSORT-B, and script filter_psort.py will filter PSORT-B outputs based on cutoff scores and localization given on parameters.

TMHMM transmembrane predicted proteins with a single helix are separated from the remaining TMHMM predictions by script filter_only_one_helix.py. Script intersect_psort_tmhmm.py compare TMHMM outputs with PSORT-B and only keeps single helix proteins (SHPs) predicted with Unknown or Membrane localization. All scripts collect the sequences of those positively filtered proteins and save them as a new .faa file in a separate folder.

Using the shortened .faa files, users must open the CMG Biotools suite to infer core proteins as described in detail below. All homologous proteins present in all strains (core proteins) will have their HLA binding affinity predicted by IEDB recommended tools using the immuno_prediction.py file. The immune_analysis.R script will combine the CMG Biotools core information with the IEDB antigenic information to discriminate protein clusters in which all homologs in all strains are highly immunogenic. It will also quantify epitope frequency per cluster (i.e., count of same peptide sequence present in proteins from a cluster) and epitope promiscuity (count of number of alleles recognized by same sequence). Comparison between epitope position and transmembrane topology can be optionally generated by the epitope_transmembrane_topology.R script.

All scripts are available in GitHub (https://github.com/fiuzatayna/epitocore). A Docker file is also available (EpitoCore_docker.tar.xz). See documentation at repository for installation and usage instructions. TMHMM, PSORT-B and CMG Biotools were run on a local desktop computer with a single Intel i7-7400 3GHz processor, 1Tb HDD and 8 Gb RAM memory. IEDB was run on a local server containing 40 cores (Intel Xeon E5-2650 2.30GHz) and 236 Gb RAM. EpitoCore pipeline performed all tasks under 4 h for *Mycobacterium avium hominissuis*.

### Data Acquisition

To compare full proteomes of *Mycobacterium avium hominissuis* strains, the amino acid sequences were obtained only for strains with complete genomes available (as November, 2018). Those are strains HP17 (GCA_002716905.1), OCU873s P7 4s (GCA_002716965.1) and OCU901s S2 2s (GCA_002716925.1) (20); H87 (GCA_001936215.1) (21), TH135 (GCA_000829075.1) (22), MAC109 (GCA_003408535.1) (23), and OCU464 (GCA_001865635.2). Each protein dataset was retrieved from the National Center for Biotechnology Information (NCBI) database using a python script that uses the Gene Assembly Summary file (assembly summary genbank.txt), available at ftp://ftp.ncbi.nlm.nih.gov/genomes/ASSEMBLY_REPORTS/. In total, each strain contained from 4,499 (OCU901s S2 2s) to 4,969 (HP17) annotated proteins.

### Identification of Transmembrane (TM) Domains

The protein datasets had alpha-helices transmembrane domains predicted by the standalone local variant of TMHMM version 2.0 (24, 25) (http://www.cbs.dtu.dk/services/TMHMM/). A second python script selected all sequences predicted to contain one or more TM alpha-helices as long as the helices comprised 18 or more amino acids. Predicted proteins were separated into two datasets: one with at least one helix occurred after the 60th N-terminal amino acid (fully embedded membrane proteins); and the other with only one helix prior to the 60th amino acid (helix near possible true signal peptide) (SHPs). Such parameters are selected accordingly to TMHMM developers' orientation. As TMHMM is a machine learning-based algorithm, such established parameters aim to reduce the number of false-positives as a result from expected pitfalls in the prediction. For example, short sequences rich in hydrophobic amino acids can be incorrectly classified as a protein containing transmembrane alpha-helices. Proteins with only one predicted helix close to protein N-terminal were further filtered as described below.

### PSORT Analysis

All sequences were evaluated using the command line version of PSORT-B version 3.0 (https://github.com/brinkmanlab/psortb_commandline_docker) (26, 27). Gram-negative parameter setup was chosen, as it considered the recommended option for gram-positive bacteria with an outer membrane, such as *Mycobacterium* spp. The remaining parameters were kept as default. Proteins predicted to be located in periplasmic or outer membrane regions are kept.

### Definition of Pangenomic Components and Surfaceome Comparison

To better predict antigens present in all strains, we first define core and accessory proteins in all proteomes using the platform Comparative Microbial Genomics (CMG) Biotools (28) version 2.2 (http://www.cbs.dtu.dk/biotools/CMGtools/). The Fasta files containing either complete proteome sequences or only predicted surfaceome entries were transferred to the platform, where CMG' pancoreplot_createConfig script was executed, followed by CMGs' pancoreplot script. CMG Biotools will consider two proteins as homologs when their BLAST alignment has at least 50% identity and 50% length coverage of the longest sequence. When BLAST aligns a protein from strain A with more than one protein in strain B, all proteins are considered homologs. We modified CMG's pancoreplot script to report and cluster (i) only the best aligned protein in strain B as an homolog; and (ii) if same result is also true when strain B is used as query, i.e., a bi-directional approach where same result is achieved for strain A and B regardless which is used as query. All homologs are then clustered and classified as a single group. CMG also aligns a protein from a strain against all proteins from same strain, so sequences from within the same strain that fulfill the alignment threshold will be clustered together, meaning that some clusters may contain more than 7 proteins. CMG Biotools then outputs a group_*n*.dat file (where *n* is the cycle number) for every strain iteration, as well as a tbl file containing a summary and other intermediary documents. The data is cumulative for every iteration, therefore we use the last group_n.dat file to select clusters with proteins present in all strains. This analysis was performed for protein datasets previous to TMHMM prediction (whole proteome), or post TMHMM prediction.

### Immunogenetic Analysis

Immunological epitope prediction was carried out using recommended methods available at the Immune Epitope Database and Analysis Resource (IEDB) (29). It is well-characterized that *Mycobacterium* species triggers MHC Class II CD4+ T cell responses in hosts (30), so we performed epitope prediction only to that MHC class. The IEDB recommended parameters for MHC-II uses the Consensus approach (31), combining NN-align, SMM-align, CombLib and Sturniolo. If no corresponding predictor is available for the allele, NetMHCIIpan is used (32). Different MHC/HLA alleles can be considered in this step. We selected 27 alleles for CD4+ T-cell epitope prediction, highly frequent in diverse populations and which were characterized as class II supertypes according to (33). The alleles selected were DRB1^*^01:01, DRB1^*^03:01, DRB1^*^04:01, DRB1^*^04:05, DRB1^*^07:01, DRB1^*^08:02, DRB1^*^09:01, DRB1^*^11:01, DRB1^*^12:01, DRB1^*^13:02, DRB1^*^15:01, DRB3^*^01:01, DRB3^*^02:02, DRB4^*^01:01, DRB5^*^01:01, DQA1^*^05:01/DQB1^*^02:01, DQA1^*^05:01/DQB1^*^03:01, DQA1^*^03:01/DQB1^*^03:02, DQA1^*^04:01/DQB1^*^04:02, DQA1^*^01:01/DQB1^*^05:01, DQA1^*^01:02/DQB1^*^06:02, DPA1^*^02:01/DPB1^*^01:01, DPA1^*^01:03/DPB1^*^02:01, DPA1^*^01/DPB1^*^04:01, DPA1^*^03:01/DPB1^*^04:02, DPA1^*^02:01/DPB1^*^05:01, and DPA1^*^02:01/DPB1^*^14:01.

For each protein, the immunogenetic prediction profile lists all different epitope-MHC allele combinations with their respective affinity scores, as well as ranked immunogenetic percentiles. Here, we defined a protein's immunogenetic Score as the mean scoring epitopes based on their percentile ranking (mean of all epitopes with score lower than 0.05). We did that for each protein within a cluster and then compared their Immunogenetic Scores—in a second filtering round, proteins whose scores were lower than 0.02 were then classified as Highly Immunogenic.

### Assigning Epitope Relevance to Protein Topology

Since the complete amino acid sequences of all predicted surfaceome proteins were submitted to epitope prediction, a vast number of highly immunogenic peptides were either present within the transmembrane region or in the intracellular portion of the molecule. Therefore, we designed a script which aligns the protein topology prediction provided by TMHMM with the epitope prediction from IEDB. Epitopes which are located fully in the intracellular or transmembrane region, or at the interface of both, are excluded from the analysis. We only considered relevant epitopes if peptides were: fully aligned to the extracellular region; or, if partially embedded in the membrane, at least more than half of the peptide should be in the extracellular region (defined as parameter outside_ratio which should be higher than 0.5).

### Validation of Predicted Epitopes

For validation of the epitopes predicted by EpitoCore, we checked if their amino acid sequences corresponded to known antigens deposited in IEDB (http://www.iedb.org/downloader.php?file_name=doc/epitope_full_v3.zip). Only antigens classified as ‘linear epitopes‘ were considered. Antigen sequences from IEDB were arranged in a fasta file using their IEDB identification as headers, and a BLAST database was created using makeblastdb script (from NCBI) for indexing. The peptides predicted as immunogenic and conserved in all seven strains (523 peptides) were then compared to the known antigens through a BLASTp-short search, which is optimized for query sequences shorter than 30 residues. Hits were evaluated using the same categories as those provided by IEDB in their “Epitope Conservancy Analysis” web application: exact matches, partial (substring) matches and according to alignment identity (90, 80, and 70%).

As a control, a dataset containing 15 mers generated by IEDB from the surfaceome proteins from all strains was created. Such dataset is exactly what EpitoCore submits to IEDB prior to epitope prediction. Identical peptide sequences between strains were reduced to a single copy in the dataset. A Monte Carlo simulation was then performed, where 527 peptides were randomly selected from the dataset and submitted to a BLASTp-short search against IEDB known antigens. The simulation was performed fifty times, and the percentage of identified antigens in every simulation was recorded.

## Results

### Protein Sequences Information and Data Analysis Layout

Even though there were 201 genome entries for the species “*Mycobacterium avium*” in the Gene Assembly Summary of NCBI as of November 2018, we opted to perform antigenic analysis only for proteomes derived from strains with complete genomes sequenced. From 18 available datasets, seven belonged to strains of the subspecies *M. avium hominissuis* and were used as the raw input data in this work. The number of protein sequences available per strain ranged from 4,499 from strain OCU901s S2 2s to 4,969 from strain HP17 (20).

**Figure 1:**
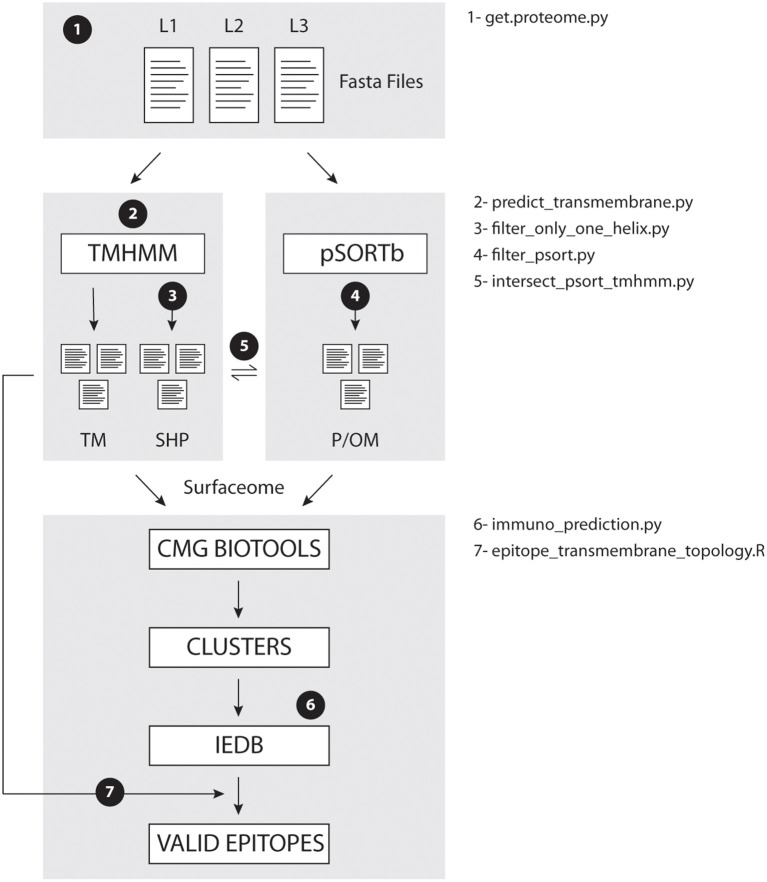
EpitoCore decision tree. Briefly, users retrieve fasta files for organism of interest, and perform transmembrane (TMHMM) and cellular localization (PSORT-B) predictions separately. TMHMM output is further divided into proteins with helix outside or not of the signal sequence. Homologs within surfaceome predictions are clustered with CMG Biotools. Core clusters had their epitopes ranked by IEDB, and that output is aligned to protein topology. Conserved epitopes in all strains are then reported. Numbers 1–7 in workflow show location where each *in house* script is called.

Figure 1 illustrates the data processing steps performed in this study. Briefly, each strain annotated proteome is submitted to TMHMM prediction and to PSORT-B prediction, to filter possible surfaceome from intracellular molecules. Surfaceome candidates are then compared across strains by CMG Biotools to define protein groups containing homologs across strains. These proteins clusters are then classified accordingly to the number of proteins present in each group. We recommend surfaceome prediction to be run before clustering because, since CMG Biotools is run on a virtual machine (not locally), performance will be faster for smaller input files. Finally, the predicted epitopes are aligned to TMHMM topology prediction, and only extracellular sequences present in most or all homologs are considered valid epitopes.

### Performance of Surfaceome and Homology Predictions

All protein entries present in the strains fasta files were submitted to transmembrane topology prediction and to cellular localization. Roughly 16% of each proteome was classified as sequences containing alpha-helix transmembrane domains or located to periplasmic and outer membrane regions (Supplementary Table 1). For now we chose to exclude beta-barrel prediction from our approach, because such method is still hampered by the limited availability of known structural data (34), and because they are mostly observed in gram-negative bacteria rather than gram-positive bacteria such as *Mycobacterium avium* (35, 36).

**Figure 2:**
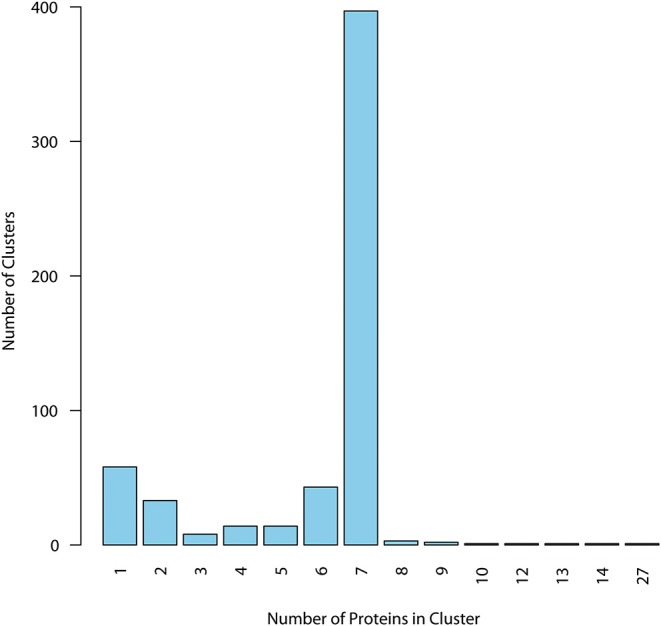
Distribution of homologs within clusters for TMHMM dataset. Proteins with membrane helix outside the signal peptide were clustered, and cluster composition was quantified. Majority of the clusters had, as expected, at least seven proteins, one from each of the analyzed strains, defining the core group. Clusters with six or less components are in principle defined as accessory proteins from the pangenome. Only core proteins were considered for immunogenic analysis.

Surfaceome protein homologs were then clustered using CMG Biotools. Ideally, we wanted to characterize epitope presence in a cluster of homologs containing one protein per strain in all strains under investigation. For simplicity, from now on we illustrate in the figures the EpitoCore performance using the TMHMM prediction as an example. But all datasets generated in each step of the protocol are given in the Supplementary Data Sheet available. Clustering of this specific dataset created a total of 577 groups, and most of those behaved as expected, i.e., the group was made of 7 proteins (397 clusters, Figure 2). Because CMG Biotools also BLAST a query entry against the remaining sequences from the same database, clusters containing more than 7 proteins were also observed. Only 10 clusters contained such possible paralogues. The remaining 170 clusters had six or less protein components and, in principle, is the accessory genome of the species. Supplementary Data Sheet 1 lists all 577 homologs from the TMHMM prediction (in green), 294 clusters classified as SHPs (in orange), and 113 periplasmic and outer membrane clusters predicted from PSORT-B (in purple). Accession numbers of the entries that belong to each group are also given.

**Figure 3:**
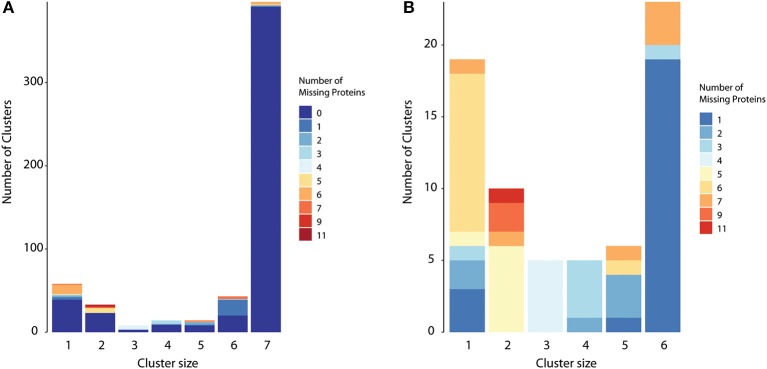
True core proteins with discrepant surfaceome prediction. To evaluate if clustering could be biased when performed on a smaller dataset, we also executed CMG Biotools in the whole proteome dataset of all strains. Protein composition for most groups was identical regardless if clustering was performed before or after surfaceome prediction (zero missing proteins when clusters are compared) **(A)**. For core proteins (column 7), 98% of the protein groups were identical. For accessory proteins (groups 1–6), this was closer to 55%. When hiding identical clusters **(B)**, it is evident that for most accessory clusters, the number of missing elements adds to the exact number of strains used. This illustrates protein groups with 7 components if no prediction is performed, i.e., true core proteins.

Some of the accessory homologs (6 or less components) might be true core proteins and were mistakenly classified due to different reasons. We were able to detect at least two issues: (i) annotation errors, meaning that the nucleotide sequence containing the gene exists in all strains, but was only annotated as a coding region in some of them; (ii) group of homologs with conflicting prediction, i.e., some members containing transmembrane helices while the remaining did not, for example. This was common in groups with lower sequence similarity between components. To demonstrate the last issue, we ran CMG Biotools in the whole proteome dataset prior to surfaceome prediction, and compared group composition in both cases. Figure 3A shows that most groups had the same composition regardless if clustered before or after the TMHMM protocol (zero missing proteins). The possible accessory proteins had observed patterns that could be explained by TMHMM variation (Figure 3B). See group 3, for example: all groups with missing proteins had exactly four missing elements, meaning that when clustered before TMHMM, all homologs in group 3 had 7 elements. All proteins in that group are then core proteins. A good number of such clusters could be re-classified as core due to different membrane domain prediction between homologs.

### MHC-II Epitope Prediction

As seen in Figure 3A, Group 7 (i.e., groups containing 7 protein sequences, one from each strain) was almost identical regardless if clustering is performed before or after any prediction. Only 6 clusters had missing proteins that were paralogues not classified as membrane proteins. For the aims of this work, epitope prediction was performed solely on clusters containing at least one homolog per strain. True core protein clusters where homologs had conflicting membrane domain or cellular localization prediction (i.e., groups 1 to 6 with missing proteins as exemplified in Figure 3B for TMHMM) were discarded.

**Figure 4:**
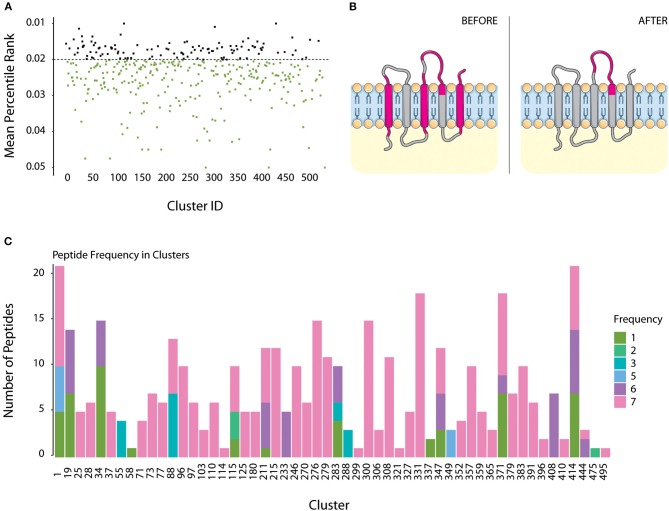
Immunogenicity Scores for TMHMM dataset and definition of valid epitopes. **(A)** All sequences within each cluster were submitted to MHC II immunogenicity scoring, to all tested alleles. Sequences with percentile score lower than 0.05 were selected. Cluster immunogenicity is given by the mean distribution of all scored epitopes under this value, for all proteins. Highly immunogenic clusters were defined as those with a mean score lower than 0.02 (black dots). **(B)** Example of a homolog from cluster 71 (Cytochrome c subunit III protein). Immunogenicity prediction defined four regions of this protein to contain possible epitopes (Before panel, in pink). As shown in the figure, three of those regions are within the transmembrane domain. Protein topology alignment allowed the removal of such regions, which were then considered invalid epitopes (After panel). **(C)** Valid epitopes were then checked if they are conserved within all homologs of its cluster. Most clusters contained conserved identical epitopes in all homologs (count 7, pink). Epitopes present in 5 or less components of the cluster were not considered for further analysis.

All sequences present in clusters with +7 components were submitted to MHC-II epitope prediction using IEDB. For each cluster, its immunogenicity score was calculated by the median distribution of its peptides percentile ranking, to any given HLA allele, but only considering the top 5% better scoring peptides. Considering only better scoring peptides, most of the TMHMM predicted core groups (387 of 407) would be considered “highly immunogenic” if a median score of 0.05 or less is used. We restricted the analysis to only a fraction of the clusters, therefore using a median score of 0.02 or less to define a highly immunogenic group. Such parameter selection defined that 112 transmembrane groups were the most immunogenic of the dataset (Figure 4A).

### Epitope Alignment to Protein Extracellular Regions

Normally, when submitting protein data for epitope prediction, the whole sequence is verified, including intracellular and transmembrane regions in transmembrane proteins. We wanted to characterize predicted epitopes which are accessible to the host immune system, i.e., located in the extracellular region of the protein. An additional filtering was then created in EpitoCore, where amino acid locations provided by TMHMM topology prediction is aligned to each predicted epitope amino acid location in same protein.

An epitope was only considered as valid if at least more than half of its length is located extracellularly. When this parameter was applied to all highly immunogenetic groups, approximately half of them were excluded because they contained no valid epitopes. Only 54 transmembrane, 34 SHPs and 15 periplasmic/outer membrane groups remained, showing that most of the predicted epitopes by IEDB are located in intracellular/transmembrane regions. Supplementary Data Sheet 1 lists the 103 highly immunogenic clusters with valid epitopes listed above. Supplementary Data Sheet 2 lists the peptide sequences of all valid epitopes to each cluster component, which alleles they trigger, position in the protein, their outside score, frequency in cluster, and promiscuity.

Figure 4B illustrates a typical example, showing topology of epitopes for a protein from cluster 71 (cytochrome c oxidase subunit 3 family protein) before and after filtering. All seven proteins from this cluster had five predicted epitope windows, four of those located in transmembrane regions (“Before” panel). Only window 120–137 (18 amino acids containing 4 overlapping 15-amino acid epitopes) was located at the surface of the cell, and is the one that is considered a valid epitope after filtering (“After” panel). Such observations are not surprising, selective pressure should be higher for sequences at the interface with the host immune system, compared to intracellular and transmembrane regions. Therefore, observations of valid epitopes in extracellular portion of a transmembrane protein should be less frequent than in regions which are not under pressure of the host immune system.

### Epitope Conservation and Validation

Overall, up to 75% of the remaining predicted epitopes are detected in at least 6 strains, while ~14% of the epitopes are unique to a single strain. Figure 4C shows epitope frequency in each of the transmembrane groups containing valid epitopes. For 35 of those, all predicted epitopes are conserved in the seven strains being evaluated. In addition, most 43 groups contained at least one epitope conserved in all strains, and 48 groups contained at least one epitope present in at least 6 strains. The remaining six clusters had epitopes which were predicted in no more than 5 strains, and those should be poor candidates for efficient immunization of all strains.

In total, there were 527 peptide epitopes predicted by EpitoCore and present in all evaluated strains. We challenged their amino acid sequences against a BLAST database created from known antigen sequences deposited in IEDB. From those, 124 peptides had sequence similarity to known antigens and are therefore considered validated epitopes (Supplementary Data Sheet 3). To guarantee that such observation is not spurious, we performed Monte Carlo simulations using a dataset from the predicted surfaceome of all strains. This dataset contains sliding 15mers created by IEDB prior to epitope prediction. Randomly sampled 527 peptides from this dataset were selected and submitted to BLASTp-short search as above, and this simulation was repeated fifty times. In all simulations, on average 7.5% of the randomly selected 15mers had sequence similarities to known antigens, with a standard deviation of 1.01 (Supplementary Figure 1).

Finally, EpitoCore is also able to calculate the minimal set of predicted epitopes potentially recognizable by the largest number of HLA alleles as possible. This calculation evaluates different combinations of epitopes with increasing group size until: (a) all alleles tested are defined or; (b) there is no gain in allele number as the number of epitopes being combined increases. With our valid epitopes dataset, we defined that 9 epitopes from 8 clusters are enough to immunize a population containing 15 of the 27 HLA alleles tested (Supplementary Data Sheet 4). Manual checking of those epitopes using BLASTp showed that none shared sequence similarity to human proteins, and four have sequence similarity to known antigens deposited in IEDB (Supplementary Figure 1). This script will work accordingly to parameters inserted by the user at previous steps. For example, if larger population coverage is desirable, user can be less stringent when defining the highly immunogenic dataset. To achieve that, the mean IEDB immunogenicity score for the top 5% of predicted epitopes can be changed to 0.05 instead of the applied 0.02. In this case, 20 clusters containing valid epitopes would be enough to trigger immunological response from 22 of the 27 tested HLA alleles (data not shown).

## Discussion

Searching for possible vaccine candidates using genomic data and bioinformatic pipelines is a well established approach (6). Nevertheless, the advances in next generation nucleotide sequencers had boosted the amount of available complete bacterial genomes. Consequently, it became evident that gene composition within genomes of strains of the same species can vary, characterizing pangenomes (12). Until now, very few studies characterizing vaccine candidates had taken pangenomic features into consideration. The majority of bioinformatics pipelines were developed to investigate single genomes, or a very limited number of strains, and so far a single pipeline has been published for pangenomic RV analysis (18).

Therefore, we implemented EpitoCore to provide not only the prediction of antigenic proteins, but also to further mine conserved peptide vaccine candidates within core proteins of a species. We tested the pipeline using seven complete genomes of *Mycobacterium avium hominissuis* strains. Once a protein cluster (i.e., homologs from analyzed strains) is scored as immunogenic, we further restricted the pipeline to filter: epitopes that are distributed equally in all homologs; correctly aligned to protein topology; triggers MHC alleles that are representative in the population; and can be defined as a minimal set of epitopes for high population coverage immunization.

Initial steps in our decision-tree workflow followed routine standards in the field, by basically eliminating intracellular and inner membrane proteins by an alpha-helix transmembrane prediction using TMHMM (24, 25) and PSORT-B (26, 27). We performed the surfaceome prediction prior to homology clustering in order to reduce dataset size and consequently, processing time. By performing homology clustering directly to the complete proteome of each strain, and comparing clusters with or without subcellular localization, we noted that many homologs had discrepant prediction due to sequence variations. While is not clear if this is taken into consideration by other publications using pangenomic features for RV, we recommend that only core proteins with similar surfaceome prediction to be considered as possible vaccine candidates.

Core homologs were then submitted to MHC II epitope prediction using IEDB (29, 37). All protein homologs that are part of a cluster are challenged. Here, presence or absence of epitopes is not enough to simply define a cluster as immunogenic. Even when using a subset of MHC alleles frequent in the population (33), practically all surfaceome proteins will have epitopes. EpitoCore lists all epitopes predicted by IEDB and sort those with lower percentile rankings (<0.05) for each protein within each cluster. The cluster immunogenicity is then given as the average percentile score of those epitopes for all proteins.

In addition to define protein groups with predicted epitopes, it is important to distinguish: (a) if epitopes are in accordance to protein topology, i.e., are located extracellularly; and (b) if epitopes are conserved amongst the homologs. For membrane proteins and SHPs, we observed that most epitopes were intracellular or within transmembrane regions, and those were discarded. Only a fraction of the clusters with epitopes predicted by IEDB could be considered to possess valid extracellular epitopes. Some of these proteins are homologs to *Mycobacterium tuberculosis* proteins known to be related to: virulence, such as mammalian cell entry proteins (clusters 710, 714, 717, 734, 753, 791,798, 836, 842, 849, and 864) (38) and type VII secretion proteins (clusters 595, 644, 773, and 774) (39); drug resistance (clusters 283, 578, 707, 722) (40, 41); drug targets (clusters 691) (42), to name a few.

Once EpitoCore defines extracellular epitopes, it will then calculate their conservation amongst homologs from same cluster and their promiscuity, i.e., if they are recognized by more than one MHC allele. Ideally, for vaccine development, epitopes present in most or all strains should be considered. Therefore, EpitoCore ranks predicted epitopes based on their frequency, and only those conserved between homologs are reported to users. Both features can be used to calculate a minimal number of epitopes which binds the largest number of MHC alleles tested. In our dataset, we determined that 9 epitopes are predicted to bind 15 of the 27 alleles tested.

Therefore, EpitoCore reported 103 groups of homologs to contain antigenic sequences. This amounts to ~2.2% of the average complete proteome (4,713 proteins) of the investigated *M. avium* strains. Such is a very conservative prediction based on performance benchmarks of open-source RV tools (6). In addition, from the predicted 527 epitopes conserved in all 7 strains, 124 (23.5%) have sequences that are similar to known antigens. Compared to simulations where 527 peptides are randomly sampled from the complete predicted surfaceome prior to epitope prediction, we showed that on average overlapping 15mers contain 7.5% known antigens. Meaning that EpitoCore has a fold-enrichment ratio of 3.13 just from the predicted surfaceome to IEDB prediction step (step 6 in Figure 1). Finally, from the 9 epitopes predicted to bind 15 of the tested MHC alleles, four are known antigens. This demonstrates that EpitoCore is successfully enriching for true epitopes from the complete proteome and surfaceome of the tested bacteria strains.

## Conclusion

EpitoCore is a peptide-centric epitope prediction tool which takes into consideration pangenomic variation across strains of a given dataset, and reports conserved epitopes between homologs of those strains. The pipeline is highly modular, and future developments could allow implementation of transcriptomics/proteomics checks to verify that homologs containing predicted epitopes are indeed expressed in the organism of interest.

## Data Availability Statement

All datasets generated for this study are included in the article/Supplementary Material.

## Author Contributions

TF designed the pipeline and evaluated the data. JL assisted with project development and discussions. GS designed the project, evaluated the data, and assisted with project development and discussions.

## Conflict of Interest

The authors declare that the research was conducted in the absence of any commercial or financial relationships that could be construed as a potential conflict of interest.

## Supplementary Material

Supplementary Figure 1 Known antigens enrichment in EpitoCore pipeline. The predicted 527 epitopes conserved in all seven strains were submitted to a BLASTp-short search against an IEDB database containing sequences from known antigens. Plot shows that 23.5% of those epitopes had sequence similarity higher than 70% with known antigens (EpitoCore). As control, we randomly sampled 527 peptides from a dataset containing 15mers peptides generated by IEDB prior to antigen check ('Surfaceome 15mers' column), and BLASTp-short searched against IEDB database as above. This sampling was performed 50 times, and the distributions of each results are shown in the plot. Overlapping 15mers contained on average 7.5% known antigens. Column 'minimun Set' represents the final 9 peptides that EpitoCore predicted to trigger antigenic response in most of the tested MHC alleles. Four of those peptides (44.4%) are known antigens deposited in IEDB.

Supplementary Table 1 Number of proteins predicted per step.

Supplementary Data Sheet 1 Protein composition of all surfaceome clusters defined by CMG Biotools (‘All clusters ID’ sheet) and those with valid epitopes predicted by EpitoCore (‘Antigenic clusters’ sheet).

Supplementary Data Sheet 2 List of all peptide sequences predicted to bind to MHC-II molecules. Sheet contains alelle bound by peptide, their IEDB scoring, protein position and protein ID, cluster ID, their calculated outside ratio, if they bind more than one alelle (promiscuity) and if are conserved in homologues (frequency).

Supplementary Data Sheet 3 List of peptide sequences predicted as epitopes by EpitoCore that match to known antigens reported by IEDB.

Supplementary Data Sheet 4 Minimal set of predicted epitopes that bind to the most number of MHC-II alelles tested.
