# Supplementary figures and images for: EpitoCore: Mining Conserved Epitope Vaccine Candidates in the Core Proteome of Multiple Bacteria Strains
Source: Front Immunol. 2020 May 5;11:816. doi: 10.3389/fimmu.2020.00816 (PMC7214623; doi:10.3389/fimmu.2020.00816)

IEDB antigens rate (%)

40

30

20

10

Surfaceome 15mers

Epitocore

minimum Set

Datasets

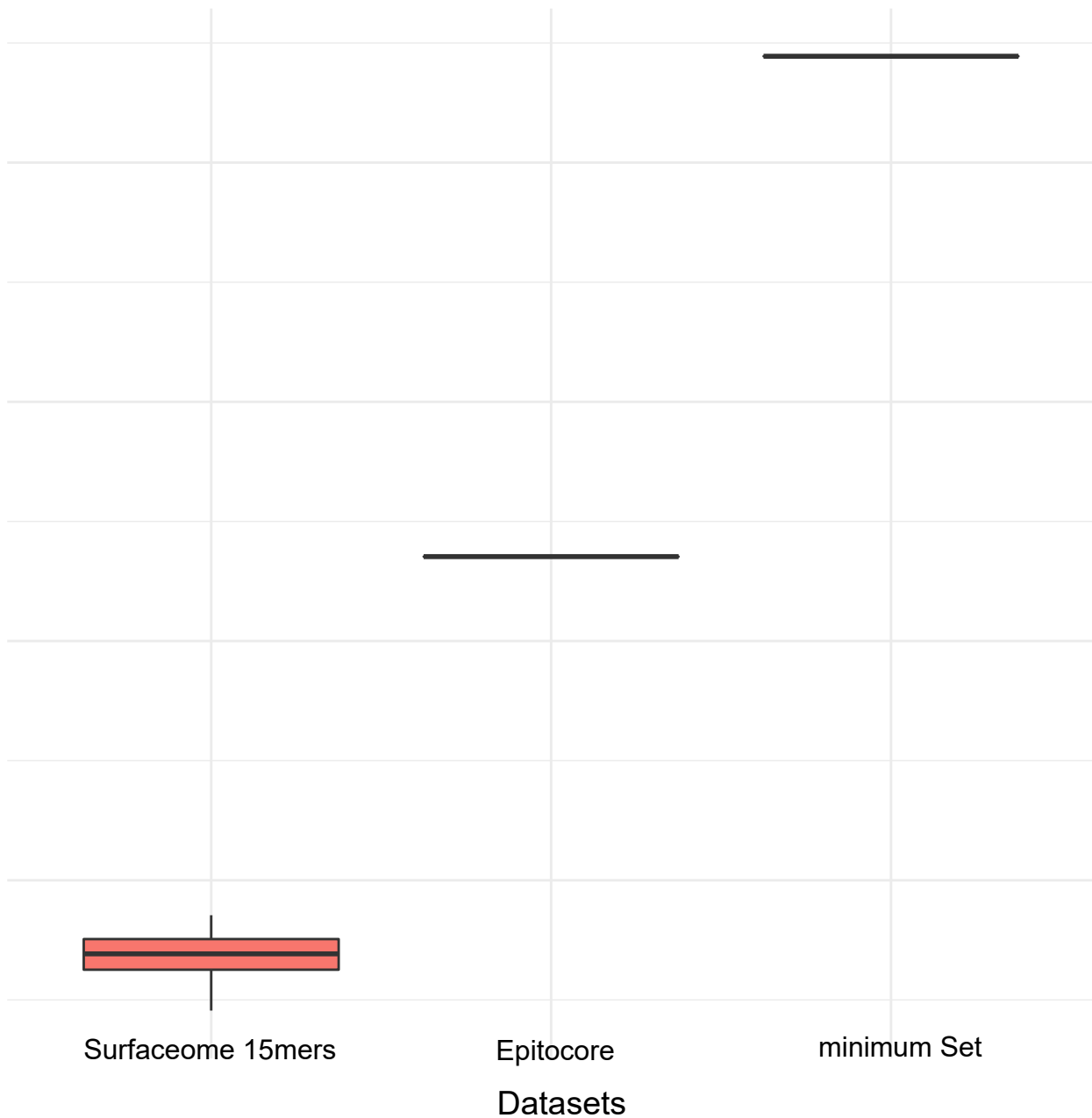

Supplement: Supplementary Figure 1 — Known antigens enrichment in EpitoCore pipeline. The predicted 527 epitopes conserved in all seven strains were submitted to a BLASTp-short search against an IEDB database containing sequences from known antigens. Plot shows that 23.5% of those epitopes had sequence similarity higher than 70% with known antigens (EpitoCore). As control, we randomly sampled 527 peptides from a dataset containing 15mers peptides generated by IEDB prior to antigen check ('Surfaceome 15mers' column), and BLASTp-short searched against IEDB database as above. This sampling was performed 50 times, and the distributions of each results are shown in the plot. Overlapping 15mers contained on average 7.5% known antigens. Column 'minimun Set' represents the final 9 peptides that EpitoCore predicted to trigger antigenic response in most of the tested MHC alleles. Four of those peptides (44.4%) are known antigens deposited in IEDB. [file Image_1.pdf]
