# Supplementary material for: EpitoCore: Mining Conserved Epitope Vaccine Candidates in the Core Proteome of Multiple Bacteria Strains
Source: Front Immunol. 2020 May 5;11:816. doi: 10.3389/fimmu.2020.00816 (PMC7214623; doi:10.3389/fimmu.2020.00816)
Supplement: Supplementary Table 1 — Number of proteins predicted per step. [file Table_1.pdf]

Supplementary Table I - Number of proteins predicted per step

STRAIN	PROTEOME	TMHMM*	SHP	PSORT
TH135	4800	481	211	83
OCU901s	4569	496	192	84
HP17	4561	490	197	87
OCU873s	4499	475	198	82
OCU464	4754	481	191	81
H87	4969	509	203	83
MAC109	4841	497	209	87

*Predicted transmembrane proteins after SHP removal
